# Detecting Mild Traumatic Brain Injury Using Resting State Magnetoencephalographic Connectivity

**DOI:** 10.1371/journal.pcbi.1004914

**Published:** 2016-12-01

**Authors:** Vasily A. Vakorin, Sam M. Doesburg, Leodante da Costa, Rakesh Jetly, Elizabeth W. Pang, Margot J. Taylor

**Affiliations:** 1 Department of Biomedical Physiology and Kinesiology, Simon Fraser University, Burnaby, British Columbia, Canada; 2 Behavioural and Cognitive Neuroscience Institute, Simon Fraser University, Burnaby, British Columbia, Canada; 3 Department of Diagnostic Imaging, Hospital for Sick Children Research Institute, Toronto, Ontario, Canada; 4 Neuroscience and Mental Health, Hospital for Sick Children Research Institute, Toronto, Ontario, Canada; 5 Department of Surgery, Division of Neurosurgery, Sunnybrook Hospital, University of Toronto, Toronto, Ontario, Canada; 6 Department of Medical Imaging, Sunnybrook Hospital, Toronto, Ontario, Canada; 7 Canadian Forces Health Services, Directorate of Mental Health, Ottawa, Ontario, Canada; 8 Department of Neurology, Hospital for Sick Children, Toronto, Ontario, Canada; 9 Department of Medical Imaging, University of Toronto, Toronto, Ontario, Canada; 10 Department of Psychology, University of Toronto, Toronto, Ontario, Canada; Universitat Pompeu Fabra, SPAIN

## Abstract

Accurate means to detect mild traumatic brain injury (mTBI) using objective and quantitative measures remain elusive. Conventional imaging typically detects no abnormalities despite post-concussive symptoms. In the present study, we recorded resting state magnetoencephalograms (MEG) from adults with mTBI and controls. Atlas-guided reconstruction of resting state activity was performed for 90 cortical and subcortical regions, and calculation of inter-regional oscillatory phase synchrony at various frequencies was performed. We demonstrate that mTBI is associated with reduced network connectivity in the delta and gamma frequency range (>30 Hz), together with increased connectivity in the slower alpha band (8–12 Hz). A similar temporal pattern was associated with correlations between network connectivity and the length of time between the injury and the MEG scan. Using such resting state MEG network synchrony we were able to detect mTBI with 88% accuracy. Classification confidence was also correlated with clinical symptom severity scores. These results provide the first evidence that imaging of MEG network connectivity, in combination with machine learning, has the potential to accurately detect and determine the severity of mTBI.

## Author Summary

Detecting concussion is typically not possible using currently clinically used brain imaging, such as MRI and CT scans. Magnetoencephalographic (MEG) imaging is able to directly measure brain activity at fast time scales, and this can be used to map how various areas of the brain interact. We recorded MEG from individuals who had suffered a concussion, as well as control subjects who had not. We found characteristic alterations of inter-regional interactions associated with concussion. Moreover, using a machine learning approach, we were able to detect concussion with 88% accuracy from MEG connectivity, and confidence of classification correlated with symptom severity. This potentially provides new quantitative and objective methods for detecting and assessing the severity of concussion using neuroimaging.

## Introduction

Detection of mild traumatic brain injury (mTBI) using neuroimaging remains a challenge, as no abnormalities are typically apparent using routine MRI [1, 2]. Accordingly, diagnosis is usually a clinical judgement based on self-report measures and behavioural assessments. Despite the lack of apparent injury on conventional clinical scans, many patients with mTBI suffer post-concussive symptoms (PCS). Although such symptoms typically resolve within a few months, a subset of individuals continue to experience long-term cognitive and behavioural impairments [3–5], underscoring the need for quantitative and objective methods for detecting and determining the severity of mTBI. The presence of lingering PCS indicates the presence of subtle brain injuries, with significant functional consequences that cannot be detected using current clinical techniques; there is a need to develop new imaging approaches for the detection of mTBI using quantitative and objective evidence.

Recent advances in magnetoencephalographic (MEG) imaging indicate that identification of mTBI is possible through detection of excessive slow-wave activity [6] and that this approach can localize the foci of the damage [7]. MTBI is associated with altered white matter microstructure as indicated by diffusion tensor imaging (DTI), in agreement with the view that mTBI results in axonal injury [8]. The focal excessive MEG slow wave activity has been shown to be related to the location of white matter injury, consistent with the supposition that oscillatory slowing can occur from deafferentation [9].

Disruption of inter-regional oscillatory synchrony in mTBI has been reported using EEG [10]. Oscillatory synchrony among brain areas is understood to play a vital role in network connectivity supporting cognition and behaviour [11, 12], and the expression of such neurophysiological network connectivity at rest relates to the intrinsic organization of brain activity pertinent for brain function and its dysfunction in clinical populations [13]. Converging evidence now indicates that traumatic brain injury is associated with diffuse axonal injury, which disrupts intrinsic functional network connectivity, thereby contributing to associated cognitive sequelae [14].

Machine learning approaches have been successfully combined with imaging of intrinsic functional brain connectivity during resting state to accurately classify single individuals [15, 16]. Moreover, electrophysiological recordings from subjects have also been shown to be effective for accurately determining group membership of individuals [17, 18]. We used MEG to investigate alterations in resting state oscillatory network synchrony in adults with mTBI, and investigated the hypothesis that machine learning algorithms could accurately detect mTBI in individual subjects.

## Results

Two groups participated in this study: adults with mTBI and adult healthy controls. Resting state MEG data were recorded, and neuromagnetic activity was reconstructed at anatomically-guided, a priori defined locations representing cortical and subcortical brain sources from the Automated Anatomical Labeling (AAL) atlas. The time-frequency representation of the source dynamics was derived using wavelet decomposition. Functional connectivity between the neuromagnetic sources was estimated in terms of frequency-specific phase-locking values. A linear support vector machine (SVM) algorithm in combination with cross-validation was applied to classify the subjects into two groups: mTBI and controls. Classification analyses were performed on different sub-sets of features, associated with different oscillatory frequencies. Classification accuracies were estimated. Associations between confidence of classification and self-reported clinical scores were investigated in the mTBI group. In addition, Partial Least Squares analysis was applied to evaluate the statistical reliability of group differences in functional connectivity.

### Detection of TBI using MEG network synchrony

**Fig 1:**
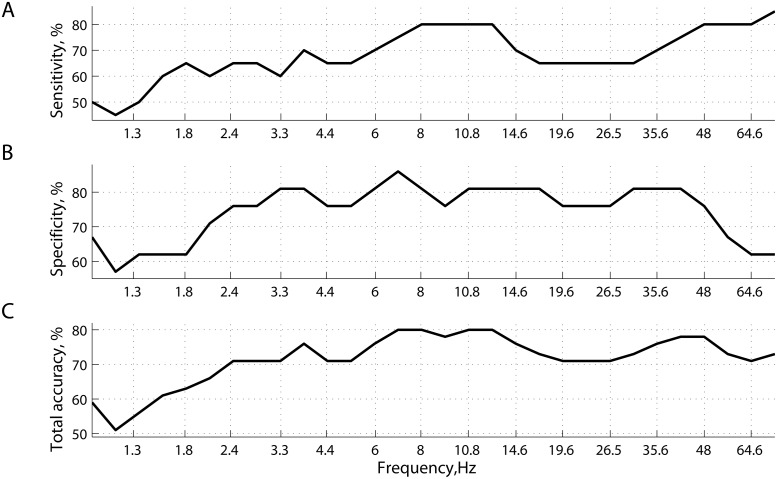
Prediction accuracy of classification. (A) sensitivity; (B) specificity; and (C) total accuracy, as functions of frequency (wavelets). Classification with cross-validation was performed separately for 30 subsets of features—connectivity matrices computed at specific frequency points.

Frequency and source resolved imaging of resting MEG network synchrony was able to accurately detect whether individual participants had been diagnosed with mTBI or not. Fig 1 shows the predictive power of phase synchrony measured at 30 specific frequencies points covering the range between 1Hz and 75Hz. Specificity remains stable around 80% and fluctuates slightly (76–83%) within a relatively wide range of frequencies: from 3Hz to 50Hz. Conversely, sensitivity and hence total accuracy have a local maximum around 8-13Hz (α rhythms), reaching 80%.

**Fig 2:**
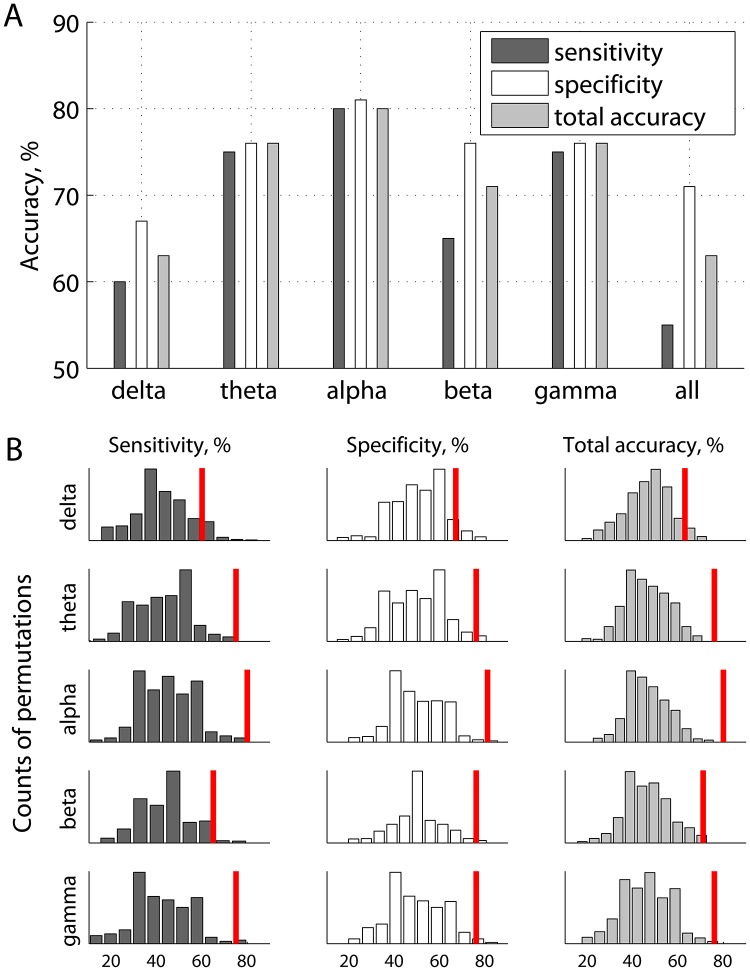
Prediction accuracies of classification as functions of frequency bands, wherein individual wavelet frequencies were grouped into 5 subsets (A), and corresponding accuracies of classification based on randomized labels representing presence or absence of mTBI (B). The red marks in panel B represent the same accuracies as in panel A.

Fig 2A provides a more aggregated view of the results shown in Fig 1. Specifically, prediction accuracy is given as functions of frequency bands, each including the features from several frequency points (wavelets). Fig 2B illustrates the same prediction accuracies with respect to the random chance prediction, wherein the distributions of accuracies were generated by shuffling the labels (mTBI or not) across subjects, and repeating the same procedure 500 times. With an accuracy of 80% (p < 0.01), inter-regional resting state phase synchrony in the α band carries the most discriminative information for inferring the presence or absence of mTBI within a single individual. Accordingly, the remainder of the results presented in this section pertain to phase synchrony in the α band, using features that individually provided the highest separability between mTBI and controls under the ROC criterion.

**Fig 3:**
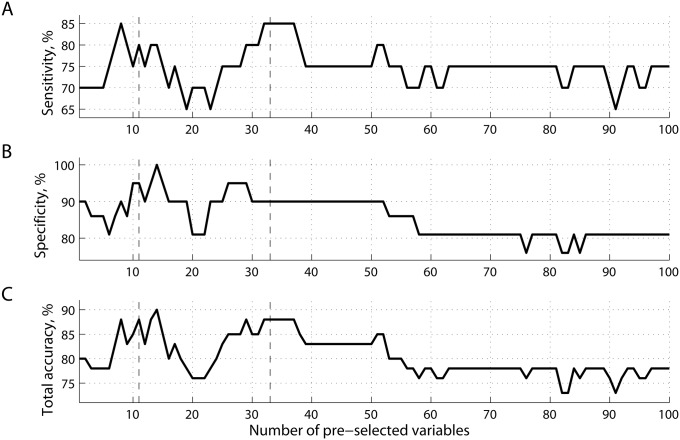
Prediction accuracy of classification. (A) sensitivity; (B) specificity; and (C) total accuracy, as functions of the number of best features ranked before training. All the features were extracted from a subset representing phase synchrony at α frequencies (see Fig 2A).

The list of ranked features reflects an estimate of how valuable a given feature was found to be for classification. We can choose the best number of features, i.e. the number that maximizes prediction accuracy. In this case, the dimensionality of the feature space will correspond to the number of source-pairings within the alpha range. Note that in this study the features were ranked using training data at each round of leave-one-out cross-validation. While the number of features to keep was set a priori, the best features themselves were determined within each round of cross-validation. Fig 3 shows accuracy values as functions of the number of best features selected for classification analysis with cross-validation. As can be seen from Fig 3C, classification accuracy can be improved with a proper threshold on the number of variables k with two peaks around k = 8−15 and k = 30−35. For example, for k = 33 accuracy is 88%, with 90% specificity and 85% sensitivity (all p < 0.01).

**Fig 4:**
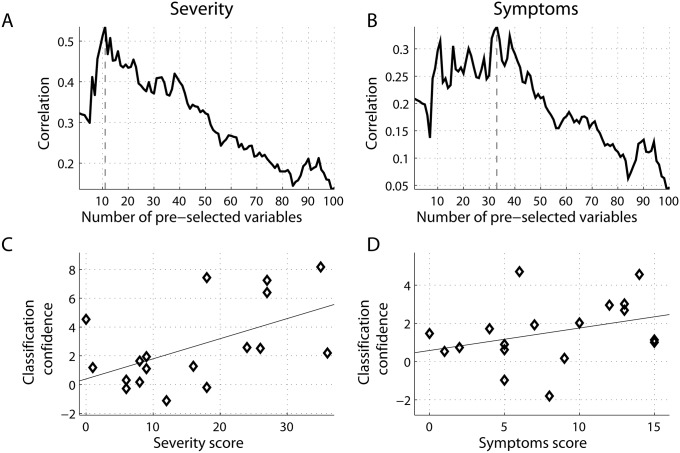
Correlations between the classification confidence (MEG-based measures) and self-reported clinical scores. (A) severity; and (B) symptoms for mTBI patients. The higher confidence that a given subject is classified as having mTBI, the higher the corresponding clinical scores. The correlations are shown as functions of the number of best features extracted from alpha connectivity and ranked before training. Scatter plots between classification confidence and clinical scores are shown at specific thresholds: k = 11 and k = 33 for (C) severity score; and (D) symptoms scores, respectively.

To investigate potential relations between SVM classification and symptom severity obtained from the concussion assessment tool (SCAT2), we quantified the distance to the decision boundary for each subject, and correlated these values with clinical scores for participants within the mTBI group. Note that the larger the distance that an individual is from the decision boundary, the higher our confidence that a subject with mTBI is classified correctly as mTBI. Similar to the procedure shown in Fig 4, for each subset of features k = 1, …, 100, we computed the distances to the decision boundary for mTBI patients, and correlated these distances with the severity and symptom scores, shown in Fig 4A and 4B, respectively. Two scatter plots with superimposed least-squares regression lines illustrate relations between these variables at two peaks, k = 11 for severity (Fig 4C), and k = 33 for symptoms (Fig 4D). Note that negative distances at the scatter plots reflect cases of misclassification, when the learning function *F*(*x*) projects the feature vectors *x* of mTBI subjects to other side of the optimal hyperplane, corresponding to controls. Moreover, the confidence of classifying a subject as mTBI positively correlated with the self-reported severity scores (Fig 4A), reaching a local maximum (r = 0.54, p < 0.05) at k = 11. It also correlated positively with the symptoms scores with a peak of r = 0.34 (p-value< 0.10) around k = 33.

**Fig 5:**
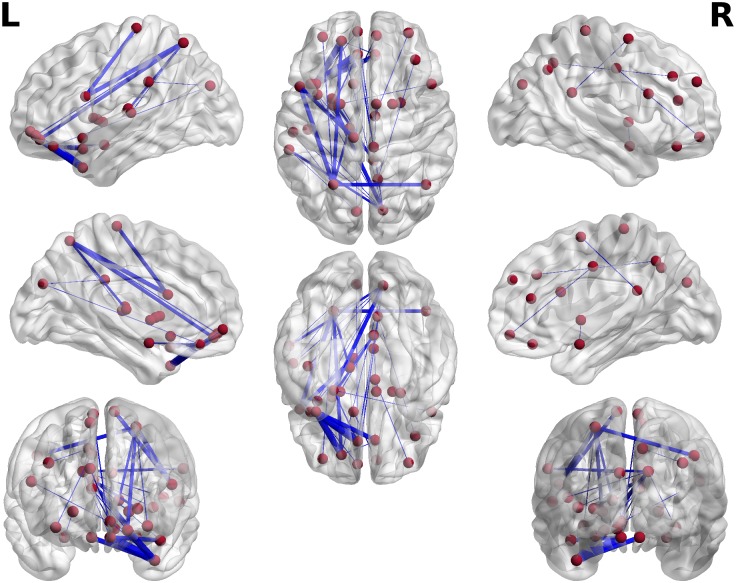
Spatial distribution of 33 best features (connectivity at alpha frequencies), which were used for prediction (see Fig 3). The red spheres indicate the location of the 33 best features (connections) for detection of mTBI, while the blue lines represent the inter-regional alpha connections (features). Line thickness denotes the robustness of contribution of specific features across subjects. The same connectiones are plotted in Fig 8B in a matrix form.

Finally, Fig 5 illustrates a distribution of the connections extracted from the pool of the best k = 33 features in the α band. It plots connections within a transparent template of the brain in the MNI space, using the BrainNet Viewer [19]. The width of the connections represents the weights are between −1 and 0, where being close to −1 implies a robust contribution of a specific connection to classification, and zero means no contribution. Specifically, for each wavelet frequency within the α range and each round of cross-validation (m = 1, …, 41), we assigned −1 to a connection if this feature survived the threshold and participated in classification, otherwise it was 0, subsequently averaging across subjects and wavelets frequencies. The ability to predict evidence of injury of a subject is largely based on synchrony between frontal and parietal/temporal sites, located mainly in the left hemisphere.

### Altered neurophysiological network connectivity in mTBI

**Fig 6:**
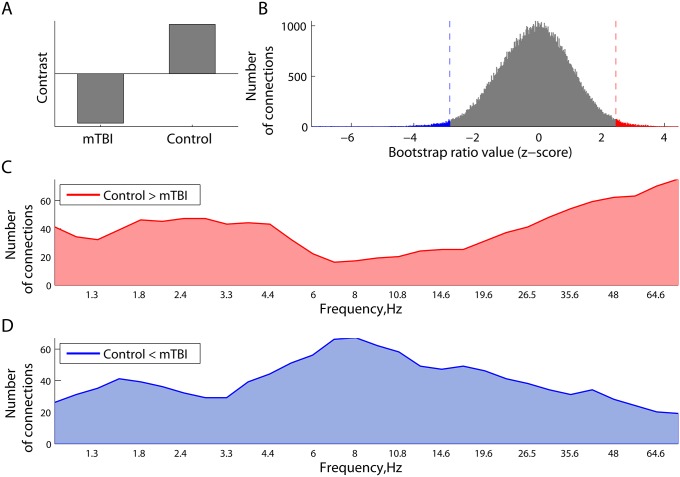
Differences in phase synchrony between the mTBI and Controls in the PLS analysis. Panel (A) illustrates the contrast between two groups. Expression of this contrast is shown in (B) as the distribution of all the bootstrap ratio values for phase-locking value between each pair of regional neuromagnetic sources at all frequencies. The distribution of these effects across frequencies, related to a decrease (C) and increase (D) in phase synchrony in mTBI participants relative to controls is plotted as the number of connections whose bootstrap ratio values belong to the corresponding 1% tails. The mTBI participants expressed reduced connectivity in the δ and γ band and hyperconnectivity at α frequencies.

We also employed PLS to characterize and test the statistical reliability of differences in resting state network synchrony between adults with and without mTBI. This analysis revealed the existence of one significant latent variable (p = 0.002) which indicated alterations of resting MEG network synchrony in mTBI (Fig 6A). The overall distribution of all the bootstrap ratio values, each associated with a pair-wise connection between the sources and frequencies, is shown in Fig 6B. As can be seen, there are relatively large positive and negative bootstrap ratio values, which reflect phase-locking and phase scattering effects, respectively, in controls with respect to mTBI.

The difference between increased and decreased phase locking is broken down further in Fig 6C and 6D, which shows how the strength of these effects varies across frequencies. Specifically, we identified the 1% tails, cut off by the 0.01− and 0.99-quantiles of the overall distribution of the bootstrap ratio values in Fig 6B. At each frequency, the number of connections with the bootstrap ratio values larger than the 0.99-quantile (right tail) was computed and plotted in Fig 6C. The strongest effects are robustly expressed at δ and lower γ frequencies, directly supporting higher phase locking in controls compared to mTBI at these frequencies. Similarly, the number of connections in the left tail defined by the 0.01-quantile is plotted in Fig 6D, as a function of frequency. These connections also support the contrast in Fig 6A, but in a reverse way, representing hyper-connectivity in the mTBI which were strongest at α frequencies.

**Fig 7:**
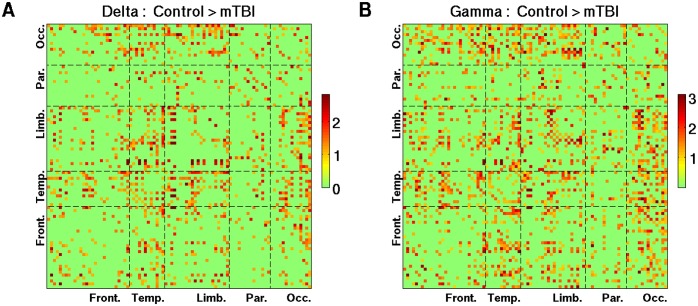
The 90 x 90 maps of the z-scores (bootstrap ratio values) reflecting higher phase synchrony in controls with respect to mTBI patients (see Fig 6C) in two canonical frequencies bands. (A) delta frequencies; and (B) gamma frequencies. Plotted are the connections associated with the bootstrap ratio values that are larger than 1.

**Fig 8:**
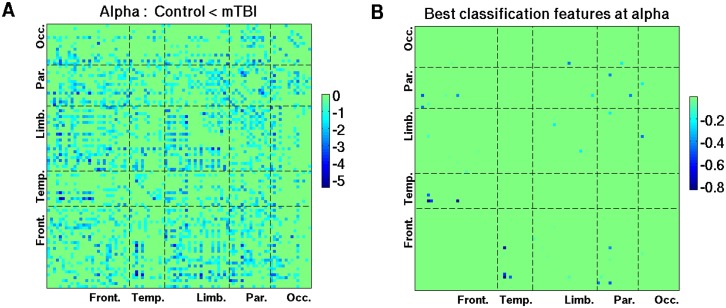
The 90 x 90 maps of increased phase synchrony at alpha frequencies in mTBI. (A) z-scores associated with the increases in connectivity in mTBI; and (B) connections cotributing to classification, robustrly across frequencies and subjects. Similar to Fig 7, panel A shows only the connections associated with the bootstrap ratio values less than -1. To compare the hyper-connectivity in mTBI at alpha frequencies with classification results, Panel B represents a matrix version of the 3D spatial pattern (Fig 5), which corresponds to the situation wherein 33 best features are used for prediction (see Fig 3).

Pair-wise connections that show decreased phase synchrony in the δ and lower γ bands in mTBI are depicted in Fig 7. The bootstrap ratio values were averaged across wavelet frequencies within corresponding frequency bands. A threshold of >1 was used for the figures to emphasize the spatial distribution. Reduced δ and γ resting phase synchrony in mTBI was most pronounced between occipital areas and other brain regions, and also preferentially involved temporal lobe connections. Similar to Figs 7 and 8A was created with a threshold of <−1, and shows the distribution of pair-wise connections associated with increased phase synchrony in mTBI at α frequencies. It is interesting to note that the distribution of connections which carry discriminative information between mTBI and controls, as illustrated on the transparent brain in the MNI space (Fig 5) and its matrix version (Fig 8B), is part of the spatial pattern representing hyper-connectivity of α rhythms in mTBI (Fig 8A), which involved numerous temporal and parietal connections.

To quantitatively compare the contribution of individual frequency bands to the contrast depicted in Fig 6A, we performed a series of steps testing difference in proportions. First, we identified the wavelets closest to the central frequencies of five canonical frequency bands: 2 Hz (delta), 6 Hz (theta), 11 Hz (alpha), 23 Hz (beta), 48 Hz (lower gamma). Then, for a given z-sore threshold (1% tails), at each central wavelet frequency, we counted connections (out of the total 90*89/2 = 4005) within the positive and negative tails of the overall distribution of *z*-scores (see Fig 6C and 6D).

For the effects defined by the tail with negative z-scores, where we observed a peak around 8 Hz (Fig 6D), we ran two-sample proportion z-tests between the alpha and other frequencies. Specifically, we tested if the numbers of connections within the negative tail at two frequency points were statistically different. We found that the number of connections was significantly higher at alpha relative to delta (p = 0.0013), beta (p = 0.0238), and lower gamma (p<0.0001), but not theta (p = 0.757).

For the positive tail of z-scores (Fig 6D), where we identified two peaks around 2 Hz and 75 Hz, we performed a series of similar two-sample proportion *z*-tests. We found that the number of connections from the positive tail was statistically higher at delta relative to theta (p = 0.0035) and alpha (0.0013), whereas the number of connections at gamma was higher than theta (p<0.001), alpha (p<0.001), and beta (0.0015), but not delta (p = 0.1211).

**Fig 9:**
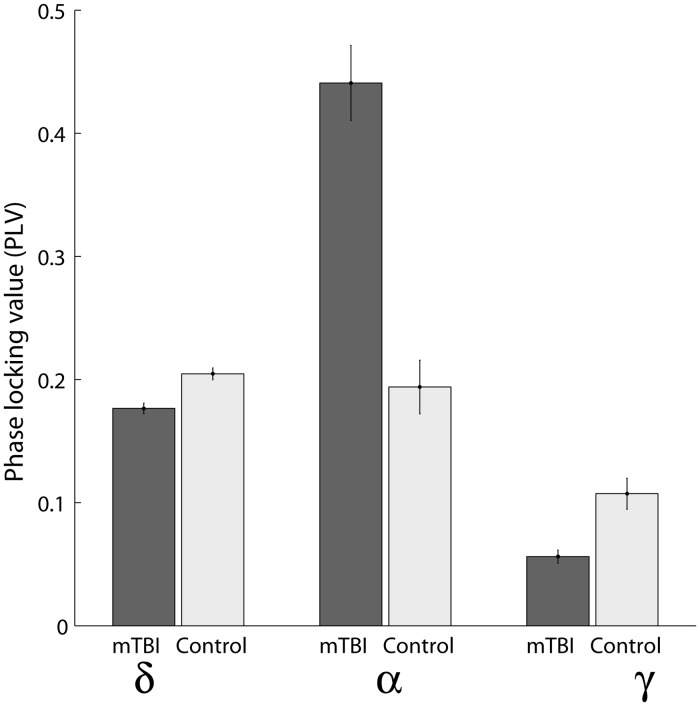
Phase-locking values for three specific connections at three characteristic frequencies. i) between the left middle occipital gyrus (Occipital Mid L) and the left median cingulate and paracingulate gyri (Cingulum Mid L) at 2 Hz; ii) between the temporal pole of the left middle temporal gyrus (Temporal Pole Mid L) and the left gyrus rectus (Rectus L) at 8 Hz; and iii) between the left inferior temporal gyrus (Temporal Inf R) and the right calcarine fissure and surrounding cortex (Calcarine R) at 75 Hz. Shown are the means across subjects and corresponding standard errors.

In addition, Fig 9 provides an example of the effects shown in Fig 6C and 6D, indicating the range of absolute values of PLV for specific connections at the characteristic frequencies. Specifically, Fig 6 depicts a spatiotemporal interplay between synchronizations and de-synchronizations in the delta, gamma, and alpha frequency bands, and we chose three connections with the largest z-scores to illustrate the effects: i) between the left middle occipital gyrus (Occipital Mid L) and the left median cingulate and paracingulate gyri (Cingulum Mid L) at 2 Hz; ii) between the temporal pole of the left middle temporal gyrus (Temporal Pole Mid L) and the left gyrus rectus (Rectus L) at 8 Hz; and iii) between the left inferior temporal gyrus (Temporal Inf R) and the right calcarine fissure and surrounding cortex (Calcarine R) at 75 Hz.

**Fig 10:**
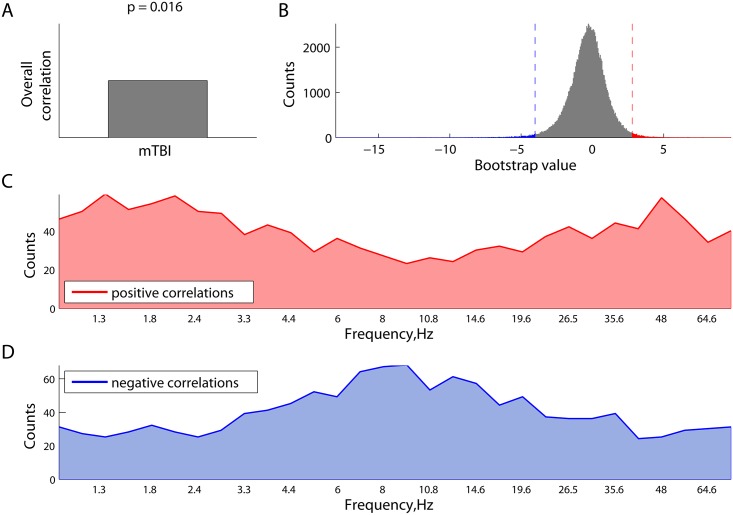
Characteristic temporal scales of the correlations between the phase synchronization and and the length of time elapsed between injury and scan, as revealed by behavioural PLS analysis. (A) overall correlation (similar to the contrast in contrast PLS); (B) overall distribution of all the bootstrap ratio values, each associated with a unique combination of frequency point and source pairing (the tails represent the most robust effects); (C) strength of the effects of positive correlations between phase-locking and time of scanning, expressed as frequency-specific number of connections from the right (red) tail in Panel B; and (D) frequency-specific number of connections from the left (blue) tail in Panel B, reflecting the negative correlations between phase synchrony and time of scanning.

Finally, we explored the effect of the length of time between injury and scan acquisition on resting MEG connectivity. We applied the behavioural PLS analysis to correlate the phase locking value with the time between brain injury and scanning. PLS analysis revealed a significant latent variable (LV) with p = 0.016, which is plotted in Fig 10A as an overall correlation (first component of LV) and a distribution of all the bootstrap ratio values (second component of LV), each associated with a unique combination of frequency and source pairing. The right (red) and left (blue) tails of the histogram in Fig 10B represent robust positive and negative correlations, respectively, between the length of time between injury and scan and phase synchronization. Frequency-specific number of connectins in these tails are shown in Fig 10C and 10D, respectively. As can be seen from Fig 10D, the effect for negative correlations between the connectivity at alpha frequencies and time of scanning is strongest at alpha frequencies. In other words, the more time that has passed since injury, the less connectivity we observed in the alpha frequency band. It is worth noting that mTBI patients, when compared to controls, were characterized by increased connectivity at alpha frequencies (Fig 6D).

## Discussion

The present study provides the first evidence for altered resting state neuromagnetic phase synchrony in a group of patients with mTBI, and showed that these alterations were associated with the amount of time elapsed between injury and scan acquisition. More importantly, we demonstrate that atypical MEG network connectivity, in combination with SVM learning, can accurately detect mTBI. This is an important step forward as mTBI is typically not detectible using conventional imaging. Our findings indicate that neurophysiological network imaging using MEG may provide an objective method for detection of mTBI. Moreover, we show that the distance of individual participants from the classification decision boundary was correlated with clinical symptom severity. These results demonstrate that MEG imaging of resting state functional connectivity may offer new approaches for assessing and tracking injury severity in mTBI.

Using a data-driven approach, we showed that group differences can be characterized in terms of interplay between synchronizations and desynchronizations at different frequencies. Specifically, we observed more increases in connectivity around theta/alpha frequencies in mTBI, whereas more decreases in connectivity in mTBI were detected for delta rhythms. This fits the hypothesis that processing of information in the brain requires both phase synchrony and phase scattering. Speculatively, phase synchronization can be viewed as a mechanism for long-range integration, whereas phase scattering can be a strategy to allow different local neural ensembles to share the same frequency channel by assigning specific neural signals to their own timeslots. Furthermore, we also found that the length of time elapsed between injury and scan tended to be negatively correlated with alpha synchronization and positively correlated with delta connectivity. These results may indicate that brain plasticity, a fundamental property for functional recovery from brain injury [20], may potentially be described in terms of redistribution of phase synchronyzation and phase scattering at different rhythms.

A similar pattern of the interplay between increases and decreases in functional connectivity was reported in an MEG study of TBI patients in two conditions: following an injury and after a rehabilitation treatment [21]. Noticeably, the study reported an opposite pattern, as increases in connectivity at higher frequencies such as alpha and beta, and conversely decreases in connectivity for delta and theta rhythms were associated with recovery from TBI. One of the key differences between the two studies was the time since injury. In our study, MEG data were recorded from mTBI patients, who were all within 3 months of injury (on average, one month). In [21], the mean time since injury was almost 4 months, and the rehabilitation program lasted for about 9 months.

Prior studies have indicated that resting state MEG can be used to detect mild and moderate TBI at the level of single individuals, but rather than focusing on inter-regional oscillatory synchrony, such research focused on the regional expression of excessive slow-wave activity [6, 7]. It has been proposed that axonal sheering caused by rapid deceleration and rotational forces plays a critical role in the pathology of TBI as well as its impact on functional networks and cognition [14]. Interestingly, regional expression of increased slow-wave activity has been shown to be either proximal to white matter abnormalities revealed by DTI, or in some cases, remote if micro-structural abnormalities occur in a major tract innervating that region [8]. Furthermore, this implies that excessive slow-wave activity reported in prior studies may be related to alterations in functional connectivity reported in the present investigation. Recent evidence indicates that regional concentrations of oscillatory slowing also correspond to particular symptoms expressed [7], raising the question of whether region-specific differences in functional connectivity may relate to specific patterns in post-concussive symptoms.

Research using EEG has also reported that electrophysiological interactions among brain regions are atypical in mTBI. Reduced inter-hemispheric phase synchrony among EEG scalp electrodes has been reported, and it was shown that such connectivity reductions in the beta and gamma frequency ranges were associated with alterations in white matter microstructure [10]. The network organization of resting state EEG connectivity has also been shown to be altered in mTBI [22]. An MEG investigation of patients with mild, moderate and severe TBI reported functional network disconnection in this group [23]. Using the data set employed in the present study, we previously showed that resting state correlations in the amplitude envelope of MEG activity is elevated in the delta, theta and alpha bands in mTBI, and that these alterations are associated with cognitive and affective sequelae in this group [24]. Interestingly, this pattern of alteration is different from MEG network alterations associated with PTSD (which is often a co-morbidity of mTBI) which was associated with high-frequency increases in resting phase synchrony [25]. Neural oscillations and their synchronization among brain areas are thought to play a critical role in cognition [11, 21], and resting neuromagnetic synchrony and amplitude correlations are presently thought to reflect intrinsic functional networks underpinning cognition, perception and their disturbance in clinical populations [13]. EEG research has also indicated that reduced electrophysiological interactions among brain areas may contribute to cognitive and behavioural problems associated with PCS. Reduced EEG coherence, for example, has been observed during visuospatial working memory in mTBI [26] and disrupted organization of network synchronization during episodic memory processing has also been reported [27]. Such reports of altered task dependent connectivity are congruent with reports of atypical electrophysiological and hemodynamic responses during cognitive processing following mTBI [28].

MRI studies have indicated altered functional network connectivity in mTBI [29–31], in the very low hemodynamic frequency oscillations measured by fMRI, which have been related to cognitive problems and recovery in this group [32]. During resting state, fMRI abnormalities have been reported which encompass visual, limbic motor and cognitive networks [29]. Altered default mode network connectivity [32] and regulation have been reported in mTBI. Spontaneous BOLD correlations have also been shown to be atypical in thalamocortical networks in mTBI patients, and these alterations are correlated with both clinical symptomatology and cognitive performance [30]. That altered connectivity is prominent in both neurophysiological and hemodynamic imaging studies is not surprising, as damage to white matter tracts in the form of diffuse axonal injury is common in severe brain injury [32–35]. Investigations of brain microstructure in such populations indicate altered axonal structure in both gray and white matter [36–38].

The present study capitalizes on rapidly emerging methods combining analysis of brain network connectivity with machine learning approaches supporting classification at the level of individual participants. This provides new insights into complex spatiotemporal shifts in intrinsic coupling in neurophysiological brain networks following mTBI. More importantly, the present work provides potentially clinically translatable methods that will permit the detection of mTBI in single individuals where conventional radiological imaging approaches are inconclusive. The finding that classification confidence is associated with self-reported symptom severity indicates that these methods may provide quantitative and objective measurements of brain changes underlying PCS. This could have significant impact on current clinical practice. An objective, quantitative method for diagnosing brain dysfunction after mTBI would allow identification of patients at risk for a subsequent injury, be invaluable for developing parameters around return to play / work / duty, and assist in developing guidelines for providing care, monitoring treatment efficacy and tracking recovery.

## Materials and Methods

### Participants

MEG data were recorded from 20 men with mTBI (21–44 years of age, mean = 31±7 years, 2 left-handed), all within three months of injury (days since injury = 32 ± 18 days). Participants with mTBI were recruited through the Emergency Department of Sunnybrook Health Science Centre in Toronto. The inclusion criteria were: concussion symptoms while in emergency; Glasgow Coma Scale ≥13 (within 24 hours of injury); if loss of consciousness occurred, then less than 30min; if post-traumatic amnesia occurred, then less than 24 hours; causes of head injury were clear (e.g., sustaining a force to the head); no skull fracture; no abnormalities on Computer Tomography (CT) scan and no previous incidences of concussion. Participants in the mTBI group completed the Sports Concussion Assessment Tool 2 (SCAT2) Symptom Checklist and Symptom Severity Score; were able to tolerate the enclosed space of the MRI; were English speaking and able to complete tasks during MEG and MR scans and able to give informed consent. The mean Severity score of mTBI patients was 20 ± 19, whereas the Symptom score was 9 ± 6. The MEG and MRI scans were obtained, on average, on 32^nd^ day since injury: 32 ± 18 days. Potential participants were screened prior to recruitment and none of the mTBI participants reported any post-traumatic stress disorder, neurological or psychiatric symptoms, and psychoactive medication use. All of the MRI scans were read by a neuroradiologist, and there were no abnormalities noted.

An age- and sex-matched control group without any history of TBI included 21 participants (20–39 years of age, mean = 27±5 years, 1 left-handed). The control group had no history of TBI (mild, moderate or severe), no neurological or psychiatric disorders, and were not on psychoactive medications. None of the participants had MRI contraindications such as metallic implants or metal dental work. Data acquisition was performed with the informed consent of each individual and with the approval of the Research Ethics Board at the Hospital for Sick Children (SickKids).

### Data acquisition

MEG data were acquired in a magnetically shielded room at SickKids using a whole-head CTF system (MISL Ltd., Coquitlam, BC, Canada) with 151 axial gradiometers as well as reference sensors for gradient correction. For each subject, 5 minutes of MEG data were continuously recorded at 600Hz using third-order spatial gradient noise cancellation. 60Hz and 120Hz notch filters were applied to MEG recordings. Data were also band-pass filtered between 1Hz and 150 Hz with a fourth-order Butterworth digital filter applied first in a forward, and then in a reverse direction so as to produce zero phase distortion. Head position during testing was monitored via three localization coils, positioned at the nasion, and the left and right pre-auricular points.

Anatomical MRI was performed on the same day at SickKids on a 3T MR scanner (MAGNETOM Tim Trio, Siemens AG, Erlangen, Germany) with a 12-channel head coil. The three fiducial coils used in the MEG were replaced with radio-opaque markers for all participants. These markers can be seen on their T1-weighted images for co-registration of the MEG source locations to the MRI images. Anatomical images were collected by whole-brain T1-weighted MRI scans (3D SAG MPRAGE: GRAPPA = 2, TR/TE/TI/FA = 2300/2.96/900/9, FOV/Res = 192x240x256, 1mm isotropic voxels).

### Reconstruction of neuromagnetic source activity

Individual MRI scans were normalized into Montreal Neurological Institute (MNI) space based on the ICBM 2009c Nonlinear Symmetric 1 × 1 × 1mm template [39]. We applied a nonlinear diffeomorphic registration, as implemented in the ANTS toolbox [40,41]. This transformation to MNI space was additionally used to warp a manually segmented inner skull surface from the MNI ICBM template to subject space. Using this inner skull surface, a multi-sphere head model was fit for each subject [42].

MEG data were co-registered to each participant’s individual anatomical MRI to constrain neuromagnetic sources to subject-specific head shape and structural anatomy. To reconstruct neuromagnetic source activity, we first selected 90 seed locations in MNI space, which represented all cortical and subcortical brain regions in the Automated Anatomical Labeling (AAL) atlas [43]. Regions specified by the AAL atlas and located in the cerebellum were excluded from the further analysis. For visualization purposes, the regions were re-ordered according to which lobe each region belongs to. The new order of the regions is given in Table 1 (the left region goes first, followed by the right one). Specifically, for each region from the AAL parcellation, the seed location was defined as a voxel within the region, which was closest, in the mean-square sense, to the means of x-, y-, and z-coordinates, averaged across all the voxels in this brain region [44]. Source estimation was performed at these 90 locations, using an adaptive spatial filter (vector beamformer) [45]. For each subject, 27 non-overlapping epochs of 10 seconds duration were extracted such that head motion within each epoch did not exceed 3mm in any direction for any of three head location coils.

**Table 1:** 

Region	*X*, mm	*Y*, mm	*Z*, mm	Lobe
Frontal Inf Orb L(R)	-37(40)	31(32)	-12(-12)	Frontal
Frontal Inf Oper L(R)	-49(49)	13(15)	19(21)	Frontal
Rectus L(R)	-6(7)	37(36)	-18(-18)	Frontal
Frontal Inf Tri L(R)	-47(49)	30(30)	14(30)	Frontal
Frontal Mid L(R)	-34(37)	33(33)	35(34)	Frontal
Frontal Mid Orb L(R)	-32(32)	50(53)	-10(-11)	Frontal
Frontal Med Orb L(R)	-6(7)	54(52)	-7(-7)	Frontal
Frontal Sup Orb L(R)	-18(17)	47(48)	-13(12)	Frontal
Frontal Sup Medial L(R)	-6(-8)	49(51)	31(30)	Frontal
Frontal Sup L(R)	-19(20)	35(35)	42(44)	Frontal
Supp Moror Area L(R)	-6(8)	5(0)	61(62)	Frontal
Precentral L(R)	-40(40)	-6(-8)	51(52)	Frontal
Rolandic Oper L(R)	-48(52)	-8(-6)	14(15)	Frontal
Insula L(R)	-36(38)	7(6)	3(2)	Frontal
Temporal Pole Mid L(R)	-37(43)	15(15)	-34(-12)	Temporal
Temporal Pol Sup L(R)	-41(47)	15(15)	-34(-32)	Temporal
Temporal Sup L(R)	-54(57)	-21(-22)	7(7)	Temporal
Temporal Mid L(R)	-57(56)	-34(-37)	-2(-1)	Temporal
Temporal Inf L(R)	-51(53)	-28(-31)	-23(-22)	Temporal
Olfactory L(R)	-9(8)	15(16)	-12(-11)	Limbic
Cingulum Ant L(R)	-5(7)	35(37)	14(16)	Limbic
Cingulum Mid L(R)	-6(7)	-15(-9)	42(40)	Limbic
Cingulum Post L(R)	-6(6)	-43(-42)	25(22)	Limbic
Hippocampus L(R)	-26(28)	-21(-20)	-10(-10)	Limbic
ParaHippocampus L(R)	-22(24)	-16(-15)	-21(-20)	Limbic
Amygdala L(R)	-24(26)	-1(1)	-17(-18)	Limbic
Caudate L(R)	-12(14)	11(12)	9(9)	Limbic
Putamen L(R)	-25(27)	4(5)	2(2)	Limbic
Pallidum L(R)	-19(20)	0(0)	0(0)	Limbic
Thalamus L(R)	-12(12)	-18(-18)	8(8)	Limbic
Paracen7tral Lobule L(R)	-9(6)	-25(-32)	70(68)	Parietal
Postcentral L(R)	-43(40)	-23(-25)	49(53)	Parietal
Parietal Sup L(R)	-24(25)	-60(-59)	59(62)	Parietal
Parietal Inf L(R)	-44(45)	-46(-46)	47(50)	Parietal
SupraMarginal L(R)	-57(5)	-34(-32)	30(34)	Parietal
Angular L(R)	-45(45)	-61(-60)	36(39)	Parietal
Precuneus L(R)	-8(9)	-56(-56)	48(44)	Parietal
Cuneus L(R)	-7(13)	-80(79)	27(28)	Occipital
Lingual L(R)	-16(15)	-68(-67)	-5(-4)	Occipital
Fusiform L(R)	-32(33)	-40(-39)	-20(-20)	Occipital
Calcarine L(R)	-8(15)	-79(-73)	6(9)	Occipital
Occipital Sup L(R)	-18(23)	-84(-81)	28(31)	Occipital
Occipital Mid L(R)	-33(36)	-81(-80)	16(19)	Occipital
Occipital Inf L(R)	-37(37)	-78(-82)	-8(-8)	Occipital

AAL-based regional map with reference to the MNI atlas.

### Phase synchronization analysis

The time-frequency representation of the original time series for each reconstructed source was derived from the wavelet decomposition, using a time-frequency toolbox [46]. Thirty frequency points equally spaced on a logarithmic scale were selected to cover the range between 1Hz and 75Hz. The analysis of phase synchronization between the neuromagnetic sources was performed on spectrally decomposed data. We computed phase-locking values [47], which are known in the literature under different names such as mean phase coherence [48] or phase synchronization index [49]. Phase synchronization emerged from studying coupled nonlinear systems [50], and is based on an idea that the existence of correlations between the phases of coupled systems does not imply correlation between their amplitudes.

A common method for obtaining phase dynamics for analyzing phase synchronization between brain signals is based on wavelet transformation [51]. A signal can be decomposed into a set of brief oscillatory patterns called wavelets. Specifically, wavelet coefficients *W*_*x*_(*τ*, *f*) at time τ and frequency *f* are obtained by convolving a given signal *x*(*t*) with a zero-mean special function or wavelet *ψ*_*τ*, *f*_(*t*):

$$W_{x}(\tau ,f)=\overset{+\infty }{\underset{-\infty }{\int }}x(t)\psi_{\tau ,f}(t)dt$$ (1)

where *ψ*_*τ*, *f*_(*t*) is a short segment of a oscillatory signal (wavelet) obtained from an elementary function called the mother wavelet by dilutions and translations. Often, a specific form of the mother wavelet is used, known as the Richer wavelet or Mexican hat function, which is defined as the negative normalized second derivative of a Gaussian function. To decompose a signal at a specific frequency *f* and time *τ*, the mother wavelet is compressed or dilated, and then translated such that *ψ*_*τ*, *f*_(*t*) is centered at time *τ*. To maintain a consistent frequency resolution, the bandwidth of the envelope is set to be inversely proportional to *f*, such that each wavelet contains the same number of cycles.

In general, the coefficients *W*_*x*_(*τ*, *f*) are complex numbers. The transformation Eq (1) thus defines both the amplitude of signal *x*(*t*) and the phase over a range of times *τ* and frequencies *f*. The instantaneous phase *ϕ*_*x*_(*τ*, *f*) is the angular component (phase angle) of *W*_*x*_(*τ*, *f*).

The relative phase Δ*ϕ*_*x*_(*τ*, *f*) of two signals, *x*(*t*) and *y*(*t*), is defined as a time series of the difference between the instantaneous phase of each signal, namely

$$\Delta \phi_{x,y}(\tau ,f)=\phi_{x}(\tau ,f)-\phi_{y}(\tau ,f)$$ (2)

which can be computed from the wavelet coefficients at time *τ* and frequency *f* from

$$e^{i\Delta \phi_{x,y}(\tau ,f)}=\frac{W_{x}(\tau ,f)W_{y}^{*}(\tau ,f)}{\left|W_{x}(\tau ,f)\right|\left|W_{y}(\tau ,f)\right|}$$ (3)

where $W_{y}^{*}(\tau ,f)$ is the complex conjugate of *W*_*y*_(*τ*, *f*).

The phase differences can be projected as a series of two-dimensional vectors onto the unit circle, one per time point *τ* = *τ*_1_, …, *τ*_*N*_. The phase-locking value *PLV*_*x*, *y*_(*f*), which reflects the amount of phase-synchrony between two signals across time, is computed as the length of the resultant (mean) vector:

$$PLV_{x,y}(f)={\langle e^{i\Delta \phi_{x,y}(\tau ,f)}\rangle }_{\tau }=\left|\frac{1}{N}\sum_{k=1}^{N}e^{i\Delta \phi_{x,y}(\tau_{k},f)}\right|$$ (4)

By construction, *PLV*_*x*, *y*_(*f*) is limited between 0 and 1. When the relative phase distribution is concentrated around the mean, the PLV is close to one, whereas phase scattering will result in a random distribution of phases and PLV close to zero.

For each epoch, for all pairs of 90 regions of interest (ROIs), frequency-specific phase differences were computed as functions of time. The phase-locking value, *PLV*_*x*, *y*_(*f*), was calculated as relative stability of the phase differences between two signals at a given frequency, subsequently averaging across epochs. Thus, 30 90-by-90 matrices were produced for each subject, representing functional connectivity in terms of phase-locking between 90 neuromagnetic sources at 30 frequency points.

### Classification

In the present study, Support Vector Machine (SVM) learning was used to predict the clinical status *Y* of a subject (mTBI or control) from a set of features *X* obtained from the subject’s MEG data [52]. These features are represented by frequency-specific phase-locking values (PLV) between the neuromagnetic activity reconstructed for 90 regions of interest (ROIs). Each of the samples (subjects) *i* = 1, …, *m*, where m = 41, can be treated as a point *x*_*i*_ in a n-dimensional feature space, where n is the total number of features—unique combinations of all the connections and frequencies of interest.

A learning machine can be seen as a function *F*, which determines a learning model:

F:X→Y (5)

The function *F* transforms vectors *x*_*i*_ from the feature domain *X* to the set *Y* of possible outcome values. When *Y* is a set of only two symbols (mTBI and control), the learning problem Eq (5) is called a binary classification, and *Y* is called the set of class labels.

Learning machines encompass many computational approaches. For classification problems, they can produce models with various types of decision borders. In this study, we applied a linear version of a SVM to determine a linear border between the classes [52]. Depending on which side of the border the sample *x*_*i*_ is located, it can be assigned to one of two classes:*Y* = {1,−1} coding mTBI and control groups, respectively. Samples used to define the border are called training data. The clinical status of new cases (test data) can be predicted based on their locations with respect to the decision border. If we know the true status of the test data, we can estimate the accuracy of that prediction. In practise, the entire data with known labels are typically divided into two sets: training data to learn the function (5) and test data to validate it.

Mathematically, learning the model (5) with a linear SVM is equivalent to finding the optimal hyperplane *ω*^*T*^*x* + *b* = 0 in the feature space, where *ω* is an n-dimensional weight vector, and–*b* defines the threshold. Optimal here means separating the two classes *Y* = {1,−1} with maximal margin. Mathematically, training the model (5) is reduced to an optimization problem, maximizing the minimum distance between vectors *x*_*i*_ and the hyperplane:

$$\underset{w,b}{\text{max}}\text{ min}\left\{\;\left\|x-x_{i}\right\|\text{ such that }w^{T}x+b=0,\;i=1,\ldots ,m\;\right\}$$ (6)

If a vector *x* satisfies *F*(*x*) = *ω*^*T*^*x* + *b* > 0, then the model (5) will assign the label 1 (class mTBI) to it, otherwise the label −1 (class Controls) is assigned. The distance from the decision boundary *F*(*x*) = *ω*^*T*^*x* + *b* can serve as a measure of confidence in the classification.

Leave-one-out cross-validation was used in this study to estimate prediction accuracies of the classification. During this procedure, all samples *x*_*i*_, *i* = 1, …, *j* − 1*j* + 1, …, *m* except one *x*_*j*_ were designated as the training data to determine the optimal model (5) for separating the classes, whereas ability of this model (5) to correctly predict the outcome was tested with the remaining sample *x*_*i*_. This procedure was repeated m times such that each subject served as the test sample only once. The prediction accuracies such as sensitivity and specificity were then computed by comparing the predicted and true statuses of m subjects.

**Fig 11:**
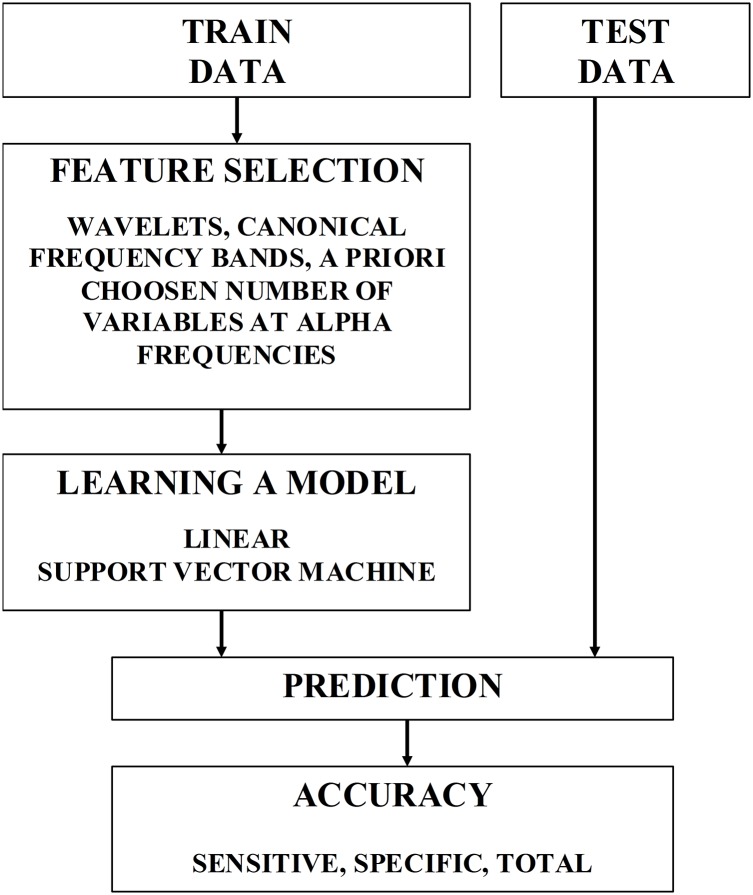
A diagram illustrating one round of leave-one-out cross-validation to estimate classification accuracies.

Until now, we assume that all features, i.e. all frequencies and connections, were used for classification. However, predictive accuracy could be improved by selecting the most relevant and informative features. In general, feature construction and selection is a critical step in classification. In practise, it is essentially heuristic. Fig 11 schematically illustrates one round of cross-validation used to learn a model from the training data, and then predict the group status of the test data. Feature selection was based on supervised learning, wherein the features were the phase synchrony estimates with three feature selection schemes: i) individual wavelet frequencies, ii) canonical frequency bands, and iii) best representative features within the α band. As a first-pass analysis, contribution of individual wavelets to the classification was estimated. Then, five frequency bands, namely δ (1-4Hz), θ (4-8Hz), α (8-14Hz), β (14-28Hz), and lower γ (28-75Hz) were a priori selected, and all the wavelets representing more fine grained frequency bins were assigned to one of these canonical bandwidths. Further, for the frequency band that carried the most discriminative information (namely, α), the features were ranked in a univariate manner. Specifically, for each feature (PLV for a given frequency and connection), overlapping probability distribution functions for two classes were compared, and the area under the resulting receiver operating characteristic (ROC) was computed [53]. The area under the ROC (AUR) provides an estimate of how valuable a feature can be for separating the two classes.

Accordingly, the feature selection can be summarized as follows. First, the features that were computed to quantify the brain state were separated into 30 sub-sets, each associated with a wavelet frequency. Classification analysis with leave-one-out cross-validation was applied separately for each subset, using linear SVM [54] as implemented in a Matlab statistics toolbox (MATLAB and Statistics Toolbox Release 2012a, The MathWorks, Inc., Natick, Massachusetts, USA). Next, total accuracy as well as specificity and sensitivity were computed. The features were then regrouped in an alternate way, and the classification process was repeated. Specifically, the features were separated into 5 subsets, each associated with a frequency band (δ, θ, α, β, lower γ), containing phase-locking values calculated for a set of wavelets within a specific frequency range. Further, the features representing phase synchrony in the α band were ranked, computing the area under the ROC curve (AUR). In the next step, leave-one-out cross-validation was applied for every number *k* = 1, …, 100 of features with the highest AUR, and classification accuracy was estimated as a function of the number of selected features. Significance of accuracy values was tested with respect to the distribution created by shuffling 500 times the labels (mTBI and Control) among the subjects.

### Partial Least Squares

Partial Least Squares (PLS) analysis was used to further explore possible group differences in connectivity (PLV) between the neuromagnetic sources across groups, as well as how these differences are expressed across frequencies and specific connections [55]. In contrast to the prediction analysis with linear SVM, wherein the learning model *F* was estimated for a subset of subjects (training data), PLS analysis was performed on the entire data in a single analysis.

PLS is a multivariate technique, which decomposes the covariance between the neurophysiological data and a discrete variable coding a contrast (between groups, for example) or a continuous variable (such as the time since injury) into mutually orthogonal factors (latent variables), similar to the principal component analysis [55]. In practice, PLS analysis can identify data-driven contrasts between groups or test specific a priori contrasts, and finds optimal relations among these contrasts and features (combinations of individual connections and frequencies in our case). Significance of the contrast can be tested with permutation tests, whereas the robustness of the contribution of specific connections and frequencies to the identified contrast can be tested with bootstrap procedures. Here we give a brief description of the technique [55–58], which was previously applied in a number of EEG and MEG studies to characterize changes in the brain signals [59–61].

PLS operates on the whole data matrix at once. Typically the rows of the data matrix correspond to participants within groups, whereas the columns correspond to voxels in functional MRI, electrodes in EEG, sensors or sources in MEG. These features (voxels, electrodes, sensors) can be called the elements. In our case, the elements were represented by all the possible combinations of a pair of neuromagnetic sources and a frequency point. Specifically, to prepare for the PLS analysis, the data matrices were organized in the form of subjects within groups by elements, each associated with a connection and a frequency point (30 × 90 × 89/2 = 120, 150 elements in total). Thus, the neuroimaging data were organized as a matrix: subjects within groups by all the possible combinations of connections and frequencies. Then, the covariances were computed between the data matrix and the vectors representing either the contrast between groups or the length of time elapsed between injury and scan.

Next, singular value decomposition (SVD) was used to project the covariances to a set of orthogonal latent variables (LVs), mathematically described as a products of three vectors: the left-singular vectors, the non-zero singular values, and the right-singular vectors. Each latent variable (LV) thus had three components: (a) a singular value, representing how much variance can be explained by this LV, similar to principal component analysis; (b) weights within the left singular vector, representing an underlying contrast between groups or an overall correlation between imaging and clinical data; (c) weights within the right singular vector (element loadings), representing the robustness of contribution of all the elements to the group contrast or overall correlations.

The overall significance of each LV and the importance of the individual elements within a specific LV was assessed using resampling procedures. First, we randomly reassigned subjects between groups, performing a permutation test. This global permutation test assessed the overall significance of a given LV, measuring how it is different from random noise. Specifically, we computed a measure of significance as the number of times the singular values from permuted data were higher than the observed singular value (500 permutations). In the second step, we tested the element loadings for stability across subjects by bootstrap resampling of subjects within groups (500 bootstrap samples). A measure of stability (bootstrap ratio value) was calculated as the ratio of the original element loading to the standard error of the distribution of the element loadings generated from bootstrapping. This is approximately equivalent to a z-score: a bootstrap ratio value of 3 or -3 corresponds to 95%-confidence under the assumption of a Gaussian distribution. Elements (all the combinations of connections and frequencies) with positive bootstrap ratio values directly support the contrast or overall correlation associated with the left-singular vector of a given LV. Negative bootstrap ratio values also indicate the robustness of the effects, but in the reverse direction. In other words, to correctly interpret the output, the bootstrap ratio values (or z-scores) need to be reported with respect to the contrast or overall correlation in order to correctly understand the direction of the loadings.

We distinguish two types of PLS analysis: so called “contrast” and “behavioural” PLS [55,56]. In the contrast PLS, there are groups of subjects (in our case, mTBI and healthy controls), and the PLV data are projected to an *a priori* defined contrast. In this case, the weights within the left singular vector are equivalent to the group contrast. The “behavioural” PLS, which is typically based only on one group of subjects, explores the covariance between the brain data and some continuous, subject-specific variables, such as time of scanning since injury. In this case, the weights within the left singular vector represent the overall correlations (one correlation per variable) between the PLV and time-of-scanning matrices. Both in the contrast and behavioural PLS, the right singular vector reflects the contribution of the individual elements to the tested effects.

### Limitations of the study

One assumption of our study is that the volume conduction effects do not represent a significant confounding factor. It is not entirely true that MEG is not sensitive to effects of volume conduction. It has been shown, however, that secondary currents resulting from volume conduction do not contribute to the radial component of the magnetic field under the assumption of a dipolar source in a spherical homogeneous conductor [62]. For our study, we used a first order axial gradiometer system, which is mainly sensitive to the radial component of the magnetic field (that is, the field of a source dipole with tangential orientation). In this setting, estimating PLV, which may capture the couplings with a phase shift close to zero, seems reasonable. Using a more conservative measure such as weighted phase lag index (PLI) would further minimize the volume conduction effects, but it would also remove some physiologically meaningful couplings, which may reduce both the sensitivity and specificity.

Another point is related to the segmentation of MEG recording. The original data were epoched into 10s segments. We choose 10s as a compromise between our intent to estimate phase synchrony at the lowest frequencies and to increase the robustness of the estimation by averaging across different epochs. Specifically, we believe that 10s is, on the one hand, long enough to robustly estimate the phase locking effects at the frequencies close to 1Hz, and on the other hand, is short enough to allow us to extract relatively large number of segments. The latter helps to increase the robustness of the results by averaging the phase synchrony across segments. Furthermore, the segments should be relatively short to not introduce large movement artefacts. Please note that the segments were extracted from 5 minutes of recordings using a rather conservative threshold of less than 3mm of movement.
